# Endometrial expression of β3 integrin, calcitonin and plexin-B1 in the window of implantation in women with unexplained infertility

**Published:** 2017-01

**Authors:** Mehran Dorostghoal, Hamid-o-allah Ghaffari, Nahid Shahbazian, Maryam Mirani

**Affiliations:** 1 *Department of Biology, Faculty of Sciences, Shahid Chamran University of Ahvaz, Ahvaz, Iran.*; 2 *Hematology, Oncology and Stem Cell Transplantation Research Center, Tehran University of Medical Sciences, Tehran, Iran.*; 3 *Department of Obstetrics and Gynecology, Imam Khomeini Hospital, Ahvaz Jundishapur University of Medical Sciences, Ahvaz, Iran.*

**Keywords:** Unexplained Infertility, Implantation, β3 integrin, Calcitonin, Plexin-B1

## Abstract

**Background:**

Endometrial receptivity plays a key role in the establishment of successful implantation and its impairment may contribute to subfertility and limit the assisted reproduction techniques (ART) success.

**Objective:**

The aim of present study was to investigate endometrial receptivity in terms of β3 integrin, calcitonin and plexin-B1 expression in women with unexplained infertility.

**Materials and Methods:**

We evaluated expression of β3 integrin, calcitonin and plexin-B1 through mRNA level measurement with real-time RT-PCR, in the endometrium of 16 infertile women with unexplained infertility and 10 fertile women. Endometrial biopsies were collected during a single menstrual cycle on postovulatory day LH+7 in each subject.

**Results:**

Significant differences regarding β3 integrin and calcitonin expression levels found between patients with unexplained infertility and the fertile women. Endometrial plexin-B1 expression levels showed no significant difference between fertile and infertile women. There were significant correlations between expression of β3 integrin with calcitonin and plexin-B1 in fertile and infertile women.

**Conclusion:**

Reduced in endometrial expression of β3 integrin and calcitonin alone or together may contribute to unexplained infertility and these genes could account as the potential molecular markers of infertility.

## Introduction

A critical step in the establishment of pregnancy in natural and assisted human reproduction is embryo implantation (1). Implantation involves complex and sophisticated molecular and cellular interactions between the blastocyst and the receptive endometrium (2). Endometrium is receptive to the embryo for a short period i.e. six days after ovulation and remains receptive for four days known as the window of implantation (3). During the menstrual cycle, several biomarkers such as hormones, receptors, cell-cell adhesion molecules, extracellular matrix proteins, growth factors, cytokines and angiogenic factors mediate morphological and physiological alterations of endometrium which allows the reception of a blastocyst and the establishment of implantation (4). Endometrial receptivity plays a key role in the establishment of successful implantation and its impairment may contribute to subfertility and limit assisted reproduction techniques (ART) success (5). 

It has been suggested that unexplained infertility is due to disturbances in molecular and cellular biomarkers involved in endometrial receptivity (6). In this regard, β3 integrin, calcitonin and plexin-B1 have been proposed as potential markers of endometrial receptivity. Endometrial β3 integrin has been identified as a cell adhesion receptor whose expression has been shown to be elevated at the time of implantation (7) and proposed as a useful marker of implantation (8). Blockade of the endometrial β3 integrin using intrauterine injection of various bioactive compounds caused implantation failure in mice (9). An association has been shown between the aberrant expression of β3 integrin and certain types of female infertility (10, 11). Calcitonin, is transiently produced in the uterine epithelia during the period of implantation (12). Attenuation of calcitonin expression during the preimplantation phase significantly decreases embryo implantation rates in rats and the administration of exogenous calcitonin could promote implantation after embryo transfer (12, 13).

It was found that calcitonin promotes the outgrowth of trophoblasts on human endometrial epithelial cell (EEC), and also, modulates the expression of certain genes in the endometrium, including down-regulating the E-cadherin expression in rodent uterine epithelium and up-regulating of β3 integrin in human endometrial carcinoma cell line (13-15). Plexin-B1 is a transmembrane semaphorines receptor implicated in the control of cell migration, angiogenesis and epithelial morphogenesis (16). Plexin-B1 is suggested to exhibit a cyclic pattern in endometrium and to have a role in endometrial receptivity (17, 18).

Evaluation of implantation markers may help to predict pregnancy outcome and detect occult implantation deficiency (19). There are still no reports for the expression of calcitonin and plexin-B1 in the pre-implantation endometrium of women with unexplained infertility. It is reported that endometrial expression of β3 integrin was lower in patients with unexplained infertility than in fertile women (8). The role of β3 integrin in implantation process is still controversial (20-24). The identification of biomarkers of endometrial receptivity has provided not only information about the molecular mechanisms underlying implantation and a means to investigate the causes of implantation failure but also a potential to be utilized these effectors for developing novel means to improve the receptivity of endometrium (5). 

Thus, present study was undertaken to investigate whether β3 integrin, calcitonin and plexin-B1 expression alter at the window of implantation in endometrium of infertile patients with unexplained infertility.

## Materials and methods

This case-control study was performed in the Laboratory of Embryology, Department of Biology, Shahid Chamran University, Ahvaz, Iran from April 2012 to October 2013. 

**Sample collection**

Endometrial samples were collected using a Novak curette in mid-luteal phase at day LH+7, from healthy volunteers women with proven fertility (n=10, age 32.5±3.2 yr) and women with unexplained infertility (n=16, age 31.6±3.0 yr) attending the hospital for treatment of infertility. Sample size was determined based on previous studies and because of the ethical considerations and difficulty in sampling was smaller in fertile group (25, 26). Concentration of luteinizing hormone (LH) in morning urine (ACON Laboratories, Inc., San Diego, USA) used to determine the day of the surge. 

All women had normal ovarian function and regular menstrual cycles with confirmation of menstrual history, and none of them had used steroid hormones for at least six months prior to the study and used intra-uterine contraceptives. Moreover, all women showed normal tubal patency and no recognizable endometriosis according to symptoms and clinical examination in transvaginal ultrasonography or diagnostic laparoscopy were seen. Women with unexplained infertility had partners with normal values of semen analysis according to the WHO criteria (27). Endometrial samples were divided into two parts. One sample was fixed in 10% formalin and embedded in paraffin. After tissue processing, 5-6 μm sections were stained with haematoxylin-eosin, evaluated histologically to correspond all samples to the assumed time in the cycle according to the Noyes *et al *criteria (28).

**Serum hormone levels**

Serum levels of luteinizing hormone (LH), follicle stimulating hormone (FSH), estradiol (E_2_) and progesterone (P_4_) were measured by the use of commercially kits (Abcam plc, Cambridge, UK).

**RNA extraction**

Total RNA were extracted from endometrial tissues (approximately 50-100mg) using Tripure (Roche Diagnostics, Germany) according to the recommended protocol by the manufacturer. RNA integrity was analyzed by electrophoresis and total RNA concentrations were obtained using a spectrophotometer at an optical density of 260 nm. The RNA was stored at -70^o^C for future procedures.

**cDNA synthesis**

Synthesis of cDNA was carried out from 1mg of total RNA from each sample with random hexamer primers using prime Script™ RT reagent Kit (Takara Bio Inc., Japan) according to the manufacturer’s instructions.

**Quantitative real-time reverse transcription polymerase chain reaction** **analysis (RT-PCR)**

Real-time RT-PCR was performed for relative quantification of the β3 integrin, calcitonin and plexin-B1 genes expression using ABI StepOne plus™ System (Applied Biosystems, Germany). Hypoxanthine phosphoribosyltransferase (HPRT) gene was used as housekeeping gene. Forward and reverse primer sequences for each gene are presented in Table І. The specificity of primers for each gene was analyzed in BLAST database (29). The reaction mixture consisted of 10µl Master mix SYBR Green, 2 µl cDNA, 1µl of each primer (10pmol/µl) and 7µl dH2O (Qiagen, Hilden, Germany). 

The standard cycling protocol used for all genes consisted of DNA denaturation and enzyme activation at 95^o^C for 10 min, denaturation 95^o^C for 15sec, annealing at 62^o^C for 15 sec and extension and florescence acquiring at 72^o^C for 15 sec. A total of 40 cycles was done. Melting curve analysis was performed by bringing temperature from 95^o^C to 60^o^C for 60 sec at the transition rate of 1 degree per second. As Livak and Schmittgen (2001) described, for sample analysis the threshold was set based on the exponential phase of products and the 2^-∆∆CT method was performed to analyze the data (30).

**Ethical Consideration**

This study was approved by the Research Ethics Committee of Shahid Chamran University of Ahvaz, Iran. Written informed consent was obtained from each participant. 

**Statistical analysis**

Data were analyzed by SPSS 16 software (SPSS Inc., USA). Independent samples student’s t-test was performed to compare characteristics and hormonal profile of the fertile and infertile women. Results are expressed as mean±SD. Comparison of β3 integrin, calcitonin and plexin-B1 expression in the groups was done using Mann-Whitney U-test. Spearman correlation analysis was carried out to investigate the relationship between variables. The level of significance was set at p<0.05.

## Results

**Figure 1:**
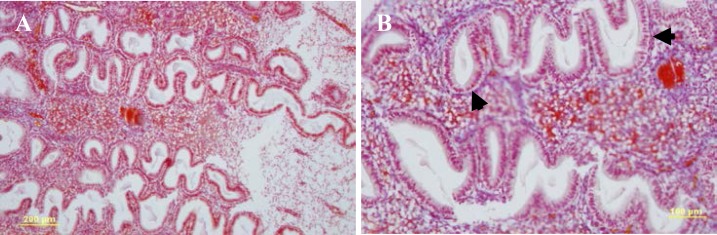
Histological sections of endometrium at mid-luteal phase (A: Scale bar=200 μm, B: Scale bar=100 μm, H&E). Stromal edema and coiled endometrial glands contain secretions with subnuclear vacuolization (black arrows) in their epithelium exhibits endometrium in mid-luteal phase

The mean age, body mass index (BMI), cycle length, duration of menses and hormonal profile in women of both groups are presented in Table ІІ. There were no significant differences in age of women, body mass index (BMI), cycle length, duration of menses and serum LH, FSH, estradiol and progesterone concentrations between two groups. Microscopic analysis of endometrial biopsies showed that all samples corresponded histologically to the mid-luteal phase of endometrial cycle (Figure 1). 

**Figure 2:**
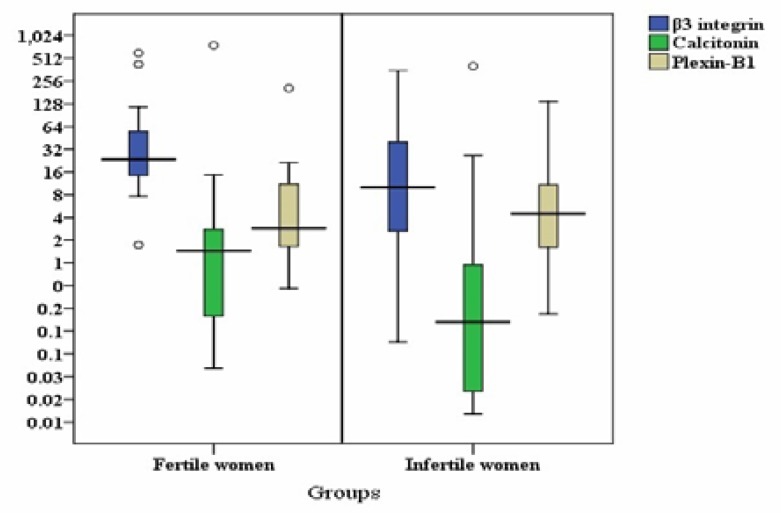
Relative expressions and co-expressions of β3 integrin, calcitonin and plexin-B1 in mid-luteal endometrium of patients with unexplained infertility (n=16) and healthy fertile women (n=10) as revealed by real-time RT-PCR

Descriptive statistics of β3 integrin, calcitonin and plexin-B1 expressions in mid-luteal endometrium of healthy fertile women and patients with unexplained infertility are shown in Table ІІІ. Figure 2 shows the relative expressions of β3 integrin, calcitonin and plexin-B1 in mid-luteal endometrium of healthy fertile women and patients with unexplained infertility. Relative quantification of β3 integrin, calcitonin and plexin-B1 mRNA is based on the expression levels of the reference gene, HPRT. 

Levels of β3 integrin mRNA expression in endometrium of patients with unexplained infertility were significantly lower than those in fertile women (p=0.043) (Figure 2). Also, calcitonin mRNA levels were significantly higher in the healthy fertile control group compared to infertile women (p=0.035) (Figure 2). While, there was no significant difference in endometrial plexin-B1 mRNA expression in patients with unexplained infertility compared to fertile women (p=0.863) (Figure 2).

**Figure 3:**
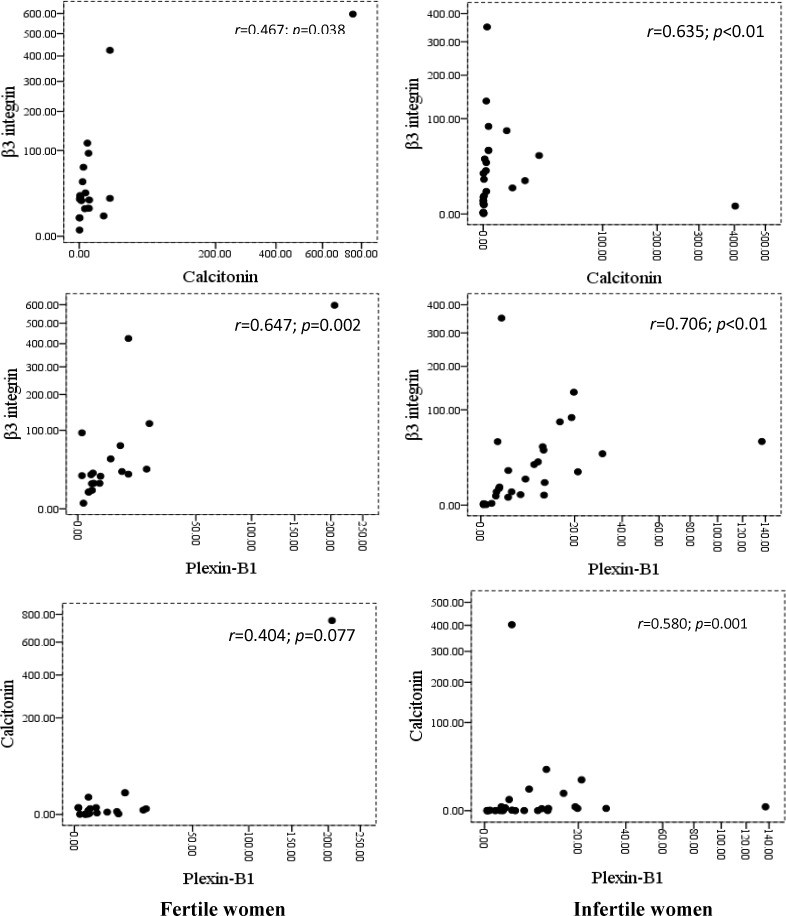
Spearman correlations between β3 integrin, calcitonin and plexin-B1 expressions in mid-luteal endometrium of healthy fertile women and patients with unexplained infertility

Statistically significant correlation was found between β3 integrin and calcitonin mRNA expression levels in fertile women (r=0.467, p=0.038) and in patients with unexplained infertility (r=0.635; p<0.01) (Figure 3). Also, there was a significant correlation between β3 integrin and plexin-B1 mRNA expression levels in the healthy fertile control group (r=0.647; p=0.002) and in infertile women (r=0.706; p<0.01) (Figure 3). A significant correlation was observed between calcitonin and plexin-B1 mRNA expression levels only in patients with unexplained infertility (r=0.580; p=0.001) (Figure 3).

**Table І:** Primer sequences used in real-time RT-PCR

**Gene**	**Forward primer(5´→3´)**	**Reverse primer(5´→3´)**	**Accession number**
β3 integrin	CATGAAGGATGATCTGTGGAGC	AATCCGCAGGTTACTGGTGAG	NM-000212
Calcitonin	TCTAAGCGGTGCGGTAATCTG	TGTTGAAGTCCTGCGTGTATG	NM-001033952
Plexin-B1	ACCAACTGCATTCACTCCCAA	GCACTCATCAGGCATCACAG	XM-011533837
HPRT	TGGACAGGACTGAACGTCTTG	CCAGCAGGTCAGCAAAGAATTTA	NM-000194

HPRT: Hypoxanthine hosphoribosyltransferase

**Table ІІ:** Characteristics and hormonal profile of the fertile and infertile women in the mid-luteal phase

**Parameter**	**Fertile women** **(n=10)**	**Infertile women** **(n=16)**	**p-value**
Age (yrs)	31.7 ±5.9	32.2±5.5	NS†
BMI (Kg/m^2^)	23.7±2.8	23.4±2.6	NS
Cycle length (days)	28.2±1.3	28.5±1.5	NS
Menses duration (days)	4.2±0.5	4.5±0.6	NS
LH (mIU/mL)	12.54±6.85	13.27±7.13	NS
FSH (mIU/mL)	5.90±2.62	6.58±2.50	NS
Estradiol (pg/ml)	139.3±55.4	142.9±61.6	NS
Progesterone (ng/mL)	10.93±3.21	11.48±4.86	NS

Independent samples t-test was done as the test of significant. Results expressed as mean±SD. The level of significance was set at *p*<0.05.

BMI: Body mass index

LH: Luteinizing hormone

FSH: Follicle stimulating hormone.

†: Non significant.

**Table ІІІ:** Descriptive statistics of β3 integrin, calcitonin and plexin-B1 expressions in mid-luteal endometrium of fertile and infertile women

**Statistics**	**β3** ** integrin**		**Calcitonin**		**Plexin-B1**	
	**Fertile**	**Infertile**	**Fertile**	**Infertile**	**Fertile**	**Infertile**
Mean	79.31	34.42	40.53	16.25	15.93	11.72
Median	23.63	8.89	1.44	0.23	2.92	3.74
SD	1.528E_2_	6.863E_1_	1.677E_2_	7.454E_1_	4.506E_1_	2.524E_1_
Q25	14.32	2.64	0.17	0.02	1.56	1.62
Q50	23.63	8.89	1.44	0.23	2.92	3.74
Q75	61.88	39.85	2.80	0.955	11.59	10.75

SD: Standard deviation

Q: Quartile.

## Discussion

A receptive endometrium plays a key role in the successful embryo implantation (31). Impaired endometrial receptivity is believed to be a considerable cause of the failure in establishment of pregnancy (32). Understanding of several endometrial growth factors, cytokines and adhesion molecules involved in the implantation might be useful for improving the endometrial receptivity to increase pregnancy rates (1). Current study undertaken to compare the expression levels of some suggested markers of endometrial receptivity in women with unexplained infertility and fertile control. 

Present study indicated that β3 integrin expression in women with unexplained infertility is impaired in the window of implantation. Healthy fertile women showed 2.3-fold higher β3 integrin mRNA expression than patients with unexplained infertility. β3 integrin proposed as a useful marker of the endometrial receptivity. During the menstrual cycle, maximal expression of β3 integrin in fertile women has been found in the implantation window (10). Blockade of the endometrial β3 integrin causes implantation failure (9). 

However, there are conflicting data about β3 integrin expression in endometrium of women with unexplained infertility. Some authors reported that there is no difference in β3 integrin expression in women with recurrent pregnancy loss (RPL) compared to fertile women (33-35). Nevertheless, Lessey *et al*, Tei *et al*, Othman *et al* and Germeyer *et al* found that women with unexplained RPL have reduced β3 integrin expression compared to controls (8, 10, 36, 37). DuQuesnay *et al* found that αvβ3 integrin mRNA expression reduced in mid-luteal phase in women with unexplained infertility (26).

Immunohistochemical studies showed that β3 integrin is reduced in patients with unexplained infertility in comparison with healthy controls (38-40). In addition, flow cytometric analysis showed that unexplained infertile women expressed lower concentrations of β3 integrin in mid-secretory phase (22). However, there are no statistically significant differences regarding αvβ3 integrin expression between infertile patients with unexplained infertility (24, 26) and endometriosis (41) compared to control women. The discrepancies that observe in expression patterns of β3 integrin could be related to technical differences.

Present study also showed down-regulation of endometrial calcitonin in the mid-luteal phase in women with unexplained infertility compared to the fertile controls. Calcitonin mRNA expression in healthy fertile group was 2.4-fold higher than infertile women. Calcitonin is expressed in the human uterine epithelium during the implantation window and has been suggested that it is one of the uterine receptivity biomarkers. It has been indicated also that the expression of calcitonin in the uterus is regulated by progesterone (12). Calcitonin may facilitate uterine receptivity by down-regulating the E-cadherin expression in rodent uterine epithelium and by inducing the tTGase expression in human endometrial epithelial cells (EECs) (15). 

Moreover, calcitonin increases the expression of β3 integrin directly and also indirectly by stimulation the heparin binding-epidermal growth factor (HB-EGF) and leukemia inhibitory factor (LIF) in human EECs (13, 41). Calcitonin regulates the functions of EECs through calcium mobilization and protein kinase C (PKC) activation (14, 15, 42). Zhu *et al* reported that intrauterine administration of antisense oligodeoxynucleotides against calcitonin mRNA during the preimplantation phase significantly decreases embryo implantation rates in rats (43). In this regard, it has been shown that the injection of calcitonin in the preimplantation phase increases the total number of implantation sites (13). Moreover, in present study levels of plexin-B1 expression in the window of implantation was lower in infertile women but the difference was not statistically significant. Harduf *et al* showed the involvement of plexin-B1 in trophoblast attachment (17). 

The correlation between plexin-B1 expression and estrogen responsiveness in breast cancer cells shows the possible involvement of steroid hormones in plexin-B1 regulation (44). Significant higher expression levels of plexin-B1 at the period corresponding to the implantation window compared to days 12-14, suggest its possible steroid regulation and role in endometrial receptivity (18). Evron *et al* reported that progesterone treatment significantly increases plexin-B1 mRNA and protein levels in endometrial cell cultures and suggested a possible role for plexin-B1 in the trophoblast-epithelial endometrial adhesion process (44). Furthermore, in present study we found positive correlations between β3 integrin, calcitonin and plexin-B1 expression at the window of implantation in both fertile and infertile women. Implantation process is a complex and multifactorial event, with association and interplay of the different factors involved. It is important to know how different markers of implantation correlate with each other (1).

It can be suggested that the positive correlations observed between β3 integrin, calcitonin and plexin-B1 expression levels could be due to both intricate interactions between these genes or similarity of factors that regulate the expression of them which remain to be explored. 

## Conclusion

In conclusion, according to our results, β3 integrin and calcitonin expression in the window of implantation reduce in women with unexplained infertility. The low expression of β3 integrin and calcitonin in women with unexplained infertility supports the possible role of these molecules in the endometrial receptivity during implantation. Therefore, these genes could account as the potential molecular markers of infertility. Plexin-B1 expression in the window of implantation does not impair in women with unexplained infertility. However, further studies are needed to determine the role of β3 integrin, calcitonin and plexin-B1 in receptivity of endometrium during the window of implantation. The identification of endometrial receptivity biomarkers and understanding the mechanisms of their relationships in endometrial receptivity may explain the phenomenon of pregnancy loss and provide new therapeutic strategies for unexplained infertility.

## Note

***This article extracted from M.Sc. thesis. (Maryam Mirani)***

## References

[1] Aghajanova L, Hamilton AE, Giudice LC (2008). Uterine receptivity to human embryonic implantation: histology, biomarkers, and transcriptomics. Semin Cell Dev Biol.

[2] Achache H, Revel A (2006). Endometrial receptivity markers, the journey to successful embryo implantation. Hum Reprod Update.

[3] Strowitzki T, Germeyer A, Popovici R, von Wolff M (2006). The human endometrium as a fertility-determining factor. Hum Reprod Update.

[4] Haouzi D, Mahmoud K, Fourar M, Bendhaou K, Dechaud H, De Vos J (2009). Identification of new biomarkers of human endometrial receptivity in the natural cycle. Hum Reprod.

[5] Cakmak H, Taylor HS (2011). Implantation failure: molecular mechanisms and clinical treatment. Hum Reprod Update.

[6] Sharkey AM, Smith SK (2003). The endometrium as a cause of implantation failure. Best Pract Res Clin Obstet Gynaecol.

[7] Illera MJ, Lorenzo PL, Gui YT, Beyler SA, Apparao KB, Lessey BA (2003). A role for alphavbeta3 integrin during implantation in the rabbit model. Biol Repod.

[8] Tei C, Maruyama T, Kuji N, Miyazaki T, Mikami M, Yoshimura Y (2003). Reduced expression of alphavbeta3 integrin in the endometrium of unexplained infertility patients with recurrent IVF-ET failures: improvement by danazol treatment. J Assist Reprod Genet.

[9] Illera MJ, Cullinan E, Gui Y, Yuan L, Beyler SA, Lessey BA (2000). Blockade of the αvβ3 integrin integrin adversely affects implantation in the mouse. Biol Reprod.

[10] Lessey BA, Castelbaum AJ, Sawin SW, Sun J (1995). Integrins as markers of uterine receptivity in women with primary unexplained infertility. Fertil Steril.

[11] Meyer WR, Castelbaum AJ, Somkuti S, Sagoskin AW, Doyle M, Harris JE (1997). Hydrosalpinges adversely affect markers of endometrial receptivity. Hum Reprod.

[12] Zhu LJ, Cullinan-Bove K, Polihronis M, Bagchi MK, Bagchi IC (1998). Calcitonin is a progesterone-regulated marker that forecasts the receptive state of endometrium during implantation. Endocrinology.

[13] Xiong T, Zhao Y, Hu D, Meng J, Wang R, Yang X (2012). Administration of calcitonin promotes blastocyst implantation in mice by up-regulating integrin b3 expression in endometrial epithelial cells. Hum Reprod.

[14] Li HY, Shen JT, Chang SP, Hsu WL, Sung YJ (2008). Calcitonin promotes outgrowth of trophoblast cells on endometrial epithelial cells: involvement of calcium mobilization and protein kinase C activation. Placenta.

[15] Li Q, Wang J, Armant DR, Bagchi MK, Bagchi IC (2002). Calcitonin down-regulates E-cadherin expression in rodent uterine epithelium during implantation. J Biol Chem.

[16] Trusolino L, Comoglio PM (2002). Scatter-factor and semaphoring receptors: cell signalling for invasive growth. Nat Rev Cancer.

[17] Harduf H, Goldman S, Shalev E (2007). Human uterine epithelial RL95-2 and HEC-1A cell-line adhesiveness: the role of plexin B1. Fertil Steril.

[18] Amir M, Romano S, Goldman S, Shalev E (2009). Plexin-B1, glycodelin and MMP7 expression in the human fallopian tube and in the endometrium. Reprod Biol.

[19] Li J, Liang X, Chen Z (2013). Improving the embryo implantation via novel molecular targets. Curr Drug Targets.

[20] Creus M, Ordi J, Fábregues F, Casamitjana R, Ferrer B, Coll E (1998). Integrin expression in normal and out-of-phase endometria. Hum Reprod.

[21] Hii LLP, Roger PAW (1998). Endometrial vascular and glandular expression of integrin αvβ3 integrin in women with and without endometriosis. Hum Reprod.

[22] Gonzalez RR, Palomino A, Boric A, Vega M, Devoto L (1999). A quantitative evaluation of β1, β4, βV and β3 endometrial integrins of fertile and unexplained infertile women during the menstrual cycle A flow cytometric appraisal. Hum Reprod.

[23] Hodivala-Dilke KM, McHugh KP, Tsakiris DA, Rayburn H, Crowley D, Ullman-Culleré M (1999). β3-integrin-deficienct mice are a model for Glanzmann thrombasthenia showing placental defects and reduced survival. J Clin Invest.

[24] Ceydeli N, Kaleli S, Calay Z, Erel CT, Akbas F, Ertungealp E (2006). Difference in αvβ3 integrin expression in endometrial stromal cell in subgroups of women with unexplained infertility. Eur J Obstet Gynecol Reprod Biol.

[25] Amjadi F, Aflatoonian R, Javanmard SH, Saifi B, Ashrafi M, Mehdizadeh M (2015). Apolipoprotein A1 as a Novel anti-implantation biomarker in polycystic ovary syndrome: A case-control study. J Res Med Sci.

[26] DuQuesnay R, Wright C, Aziz AA, Stamp GW, Trew GH, Margara RA (2009). Infertile women with isolated polycystic ovaries are deficient in endometrial expression of osteopontin but not alphavbeta3 integrin during the implantation window. Fertil Steril.

[27] WHO (2010). Laboratory Manual for the Examination of Human Semen and Sperm-cervical Mucus Interaction.

[28] Noyes RW, Hertig AT, Rock J (1975). Dating the endometrial biopsy. Am J Obstet Gynecol.

[29] Ye J, Coulouris G, Zaretskaya I, Cutcutache I, Rozen S, Madden TL (2012). Primer-BLAST: A tool to design target-specific primers for polymerase chain reaction. BMC Bioinformatics.

[30] Livak KJ, Schmittgen TD (2001). Analysis of relative gene expression data using real-time quantitative PCR and the 2-DDCT method. Methods.

[31] Revel A (2012). Defective endometrial receptivity. Fertil Steril.

[32] Macklon NS, Stouffer RL, Giudice LC, Fauser BC (2006). The science behind 25 years of ovarian stimulation for in vitro fertilization. Endocr Rev.

[33] Tuckerman EM, Laird SM, Prakash A, Li TC (2006). Expression of integrins in the endometrium of women with recurrent miscarriage. Fertil Steril.

[34] Xu B, Sun X, Li L, Wu L, Zhang A, Feng Y (2012). Pinopodes, leukemia inhibitory factor, integrin-beta3, and mucin-1 expression in the peri-implantation endometrium of women with unexplained recurrent pregnancy loss. Fertil Steril.

[35] Coughlan C, Sinagra M, Ledger W, Li TC, Laird S (2013). Endometrial integrin expression in women with recurrent implantation failure after in vitro fertilization and its relationship to pregnancy outcome. Fertil Steril.

[36] Othman R, Omar MH, Shan LP, Shafiee MN, Jamal R, Mokhtar NM (2012). Microarray profiling of secretory-phase endometrium from patients with recurrent miscarriage. Reprod Biol.

[37] Germeyer A, Savaris RF, Jauckus J, Lessey B (2014). Endometrial beta3 integrin profile reflects endometrial receptivity defects in women with unexplained recurrent pregnancy loss. Reprod Biol Endocrinol.

[38] Franasiak JM, Holoch KJ, Yuan L, Schammel DP, Young SL, Lessey BA (2014). Prospective assessment of midsecretory endometrial leukemia inhibitor factor expression versus ανβ3 testing in women with unexplained infertility. Fertil Steril.

[39] Boroujerdnia MG, Nikbakht R (2008). Beta3 integrin expression within uterine endometrium and its relationship with unexplained infertility. Pak J Biol Sci.

[40] Chen D, Jin Z, Xing F (1998). The expression of integrin beta 3 in cycling and early pregnant endometrium and its relationship with primary unexplained infertility. Zhonghua Fu Chan Ke Za Zhi.

[41] Casals G, Ordi J, Creus M, Fábregues F, Carmona F, Casamitjana R (2012). Expression pattern of osteopontin and αvβ3 integrin during the implantation window in infertile patients with early stages of endometriosis. Hum Reprod.

[42] Li Q, Bagchi MK, Bagchi IC (2006). Identification of a signaling pathway involving progesterone receptor, calcitonin, and tissue tranglutaminase in Ishikawa endometrial cells. Endocrinology.

[43] Zhu LJ, Bagchi MK, Bagchi IC (1998). Attenuation of calcitonin gene expression in pregnant rat uterus leads to a block in embryonic implantation. Endocrinology.

[44] Evron A, Goldman S, Shalev E (2011). Effect of primary human endometrial stromal cells on epithelial cell receptivity and protein expression is dependent on menstrual cycle stage. Hum Reprod.

